# Nanotechnology Applications in Sepsis: Essential Knowledge for Clinicians

**DOI:** 10.3390/pharmaceutics15061682

**Published:** 2023-06-08

**Authors:** Inês Vasconcelos, Tiago Santos

**Affiliations:** 1 School of Medicine, University of Minho, 4710-057 Braga, Portugal; 2 Department of Surgery and Physiology, Cardiovascular Research and Development Center—UnIC, Faculty of Medicine, University of Porto, Al. Prof. Hernâni Monteiro, 4200-319 Porto, Portugal

**Keywords:** sepsis, diagnostic, treatment, drug delivery, nanomedicine, nanotechnology

## Abstract

Sepsis is a life-threatening condition caused by a dysregulated host response to an invading pathogen such as multidrug-resistant bacteria. Despite recent advancements, sepsis is a leading cause of morbidity and mortality, resulting in a significant global impact and burden. This condition affects all age groups, with clinical outcomes mainly depending on a timely diagnosis and appropriate early therapeutic intervention. Because of the unique features of nanosized systems, there is a growing interest in developing and designing novel solutions. Nanoscale-engineered materials allow a targeted and controlled release of bioactive agents, resulting in improved efficacy with minimal side effects. Additionally, nanoparticle-based sensors provide a quicker and more reliable alternative to conventional diagnostic methods for identifying infection and organ dysfunction. Despite recent advancements, fundamental nanotechnology principles are often presented in technical formats that presuppose advanced chemistry, physics, and engineering knowledge. Consequently, clinicians may not grasp the underlying science, hindering interdisciplinary collaborations and successful translation from bench to bedside. In this review, we abridge some of the most recent and most promising nanotechnology-based solutions for sepsis diagnosis and management using an intelligible format to stimulate a seamless collaboration between engineers, scientists, and clinicians.

## 1. Introduction

Sepsis is a clinical syndrome characterized by physiological, pathological, and biochemical abnormalities induced by an invading pathogen, causing dysregulated host immune response and resulting as ultimately responsible for life-threatening organ dysfunction. It is a leading cause of morbidity and mortality, affecting all age groups and representing a significant global burden [1,2,3]. Clinical outcomes in patients admitted due to sepsis mainly depend on timely diagnosis and appropriate early therapeutic intervention.

Various consensus meetings have been held in recent decades to better define sepsis as a clinical entity. In the 1990s, sepsis was characterized by a systemic inflammatory response syndrome that, when complicated by organ dysfunction, was termed “severe sepsis” and could progress to septic shock [4,5]. Despite the limitations of these definitions and the attempts to identify precise diagnostic criteria, the definitions of sepsis, septic shock, and organ dysfunction have remained mainly unchanged. To unify these concepts, the Sepsis-3 meeting recently defined sepsis as a life-threatening organ dysfunction caused by a dysregulated host response to infection, emphasizing the primacy of a nonhomeostatic host response, the potential lethality, and the need for early recognition [5]. Even a modest degree of organ dysfunction is associated with an in-hospital mortality excess of 10% [5]. Despite advancements in the understanding of sepsis as a clinical entity and its pathophysiology, it remains a common condition with no approved specific molecular therapies and significant mortality [6,7,8]. Controversy continues to surround nearly every variable in the management of sepsis. At the same time, clinical trials fail to show significant results in the attempts to normalize or enhance various aspects of the physiology of these patients [9].

Nanotechnology is considered one of the most promising technologies of the 21st century and refers to the design and use of technologies at the nanoscale. These structures have unique physical, chemical, and biological properties that can be of interest in the engineering of devices and diagnostic systems, as well as for treating some medical conditions. The possibility to design nanostructures with specific characteristics such as size, shape, elasticity, surface charge, and functionalization allows its application in biomedical areas ranging from drug delivery, vaccine, and antibacterial drug development to diagnosis and imaging tools [10,11]. The in-depth nanotechnological principles and modulation of these materials’ physicochemical properties are beyond the scope of this review; however, they have been covered by other authors [12,13].

Due to the versatility of these materials, there is growing interest in their application in the medical field, particularly in the management of sepsis [14]. In recent years, innovative nanoparticle-based sensors have been explored for the point-of-care identification of infections and organ dysfunction [15,16]. Nanoscale-engineered materials allow a controlled and efficient release of bioactive agents to target organs and cells, resulting in improved efficacy with currently available drugs having minimal side effects [17]. Despite promising results, clinical translation remains intricate. The fundamental principles of nanotechnology are often presented in highly technical formats that are difficult for the non-expert to comprehend. This ultimately hinders collaborations with clinicians and delays the translation of nanotechnological approaches to the patient bedside. With this review, we aim to introduce some of the most recent and most promising outcomes of nanotechnology applications to the field of sepsis in an accessible format for both clinicians and the scientific community.

## 2. Pathophysiology of Sepsis

Sepsis is fundamentally an inflammatory disease consisting of short-term hyperinflammatory and longer-term immunosuppressive phases [17]. In an early establishment phase, a “cytokine storm” induces an overwhelming inflammatory reaction, resulting in high fevers and refractory shock that can be followed by cardiac and pulmonary failure [17]. This initial phase of the disease is responsible for the highest death rates. After that, exhaustion of the immune system, immune cell dysfunction, and apoptosis cause persistent immunosuppression, resulting in organ damage and failure and late-period mortality [18].

The triggering event is the recognition of pathogen-associated molecular patterns (PAMPs) or danger-associated molecular patterns (DAMPs). The recognition of these and other microbial-derived products by epithelial and endothelial cell populations triggers a complex intracellular signaling system that promotes inflammation. The microbially derived molecules and the signaling pathways activated regulate the intensity and direction of the inflammatory response. Simultaneously, the activation of the complement system and production of pro-inflammatory cytokines profoundly affects coagulation and vascular endothelium function. During the establishment of sepsis, the expression of adhesion molecules, including pro-coagulant and anti-coagulant proteins, is significantly altered, resulting in the transition of the epithelium from an anti-coagulant to a pro-coagulant state. The overactivation of complement system mediators is also associated with the generation of elevated levels of reactive oxygen species and the release of granular enzymes, causing significant inflammatory tissue damage. These mechanisms are believed to contribute to vasodilation, tissue damage, and multiple organ failure in acute infection (as reviewed in [19]).

Although early systemic inflammatory response is considered a hallmark of sepsis, immunosuppression is usually observed in these patients. Surviving patients exhibit persistent inflammation/immunosuppression and catabolism syndrome [20,21]. The main features of this clinical syndrome are markedly increased C-reactive protein (CRP) concentrations, neutrophilia, and the release of immature myeloid cells [22]. Immature myeloid cells have defective antimicrobial activity and produce anti-inflammatory cytokines when mobilized to circulation, downregulating the inflammation and resulting in functional immunosuppression. Although the etiology of this entity is unknown, it is likely driven by DAMPs produced by injured tissues and organs [23].

## 3. Nanotechnology at a Glance

Nanotechnology is a complex field encompassing solid-state physics, materials science, surface chemistry, and quantum mechanics. Although the underlying science is complex and often presented in highly technical formats, we aim to distill the main concepts required for a working knowledge of nanoscience and an appreciation of its potential as a clinical tool.

Nanomaterials have a characteristic dimension from 1 to 100 nm and are generally classified into organic and inorganic materials. These are designed with specific chemical, physical, and surface properties that yield the desired biological properties and functions [12]. Depending on the materials used, adding or subtracting a few atoms can significantly impact the size and shape of the structure and, consequently, its effects. Nanomaterials can transport drugs by adsorbing, entrapping, or binding covalently to them.

**Figure 1:**
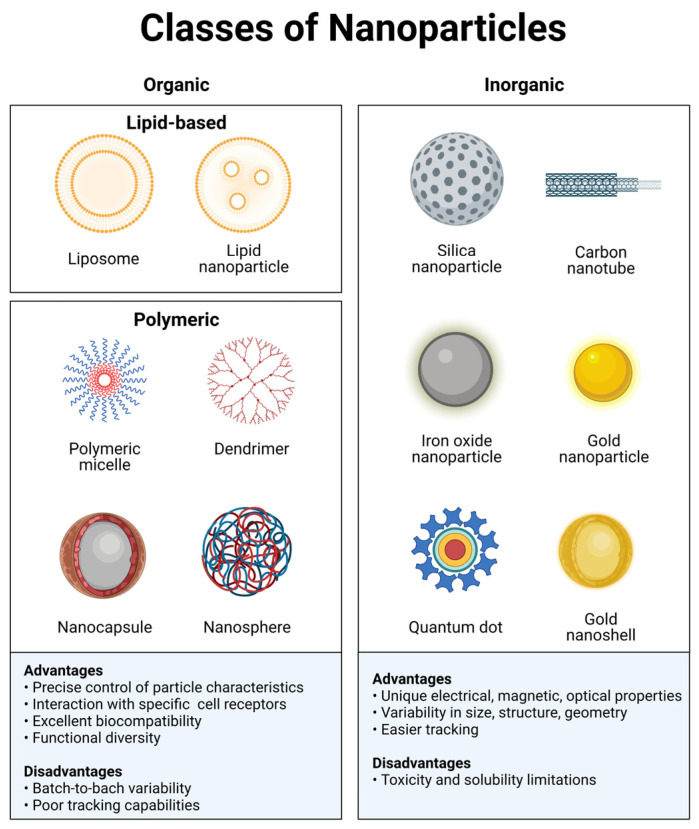
Classes of nanoparticles. Each class has numerous advantages and disadvantages regarding cargo, delivery, and patient response. Image created with BioRender.com.

Organic nanomaterials typically comprise carbonated skeletons that can either be lipid-based or synthetic polymeric materials (Figure 1). Some examples of organic materials are: protein-based, polysaccharides, chitosan, liposomes, polymeric micelles, poly(ethylenimine), poly(alkylcyanoacrylates), poly(amidoamine) dendrimers, or poly(lactic-co-glycolic acid). These materials exhibit excellent biocompatibility and low toxicity and do not elicit significant immunological responses since they mainly consist of carbon, nitrogen, and oxygen. An additional advantage of organic nanomaterials, particularly biologically derived ones, is the interaction with specific receptors/transporters [24]. Organic nanomaterials have great functional diversity, and their chemical and physical properties can be modulated to carry medical agents and favor binding to a particular subset of cells. Researchers can modulate the composition of organic nanoparticles through the conjugation of molecules, such as antibodies or peptides. This functionalization of the material allows interaction with a diverse range of biological moieties to achieve targeted delivery. A disadvantage of these materials is the batch-to-batch variability, limited ability for controlled modification, and poor tracking capabilities. Organic nanomaterials are currently being used to develop vaccines, immunotherapy, and diagnostics.

Inorganic materials include metals such as gold, copper, zinc, and aluminum; semiconductors such as cadmium selenide, zinc oxide, and carbon nanotubes; and compounds such as iron oxide or calcium phosphate (Figure 1). An exciting feature of these particles is their tunable properties. The electrical, optical, and magnetic properties can be modulated by changing their physicochemical design. Inorganic nanoparticles offer advantages such as a better control over their size and shape and a simplicity of preparation and functionalization [24]. Most importantly, these particles are generally easier to track by either microscopic or analytic techniques. However, their stability, biocompatibility, and immunogenicity are less favorable than in organic ones. Nonetheless, a significant amount of research has been dedicated to overcome this problem. These issues can be minimized by either coating or encapsulating them with biocompatible materials [12].

Some of the main features influencing delivery and function besides nanoparticle composition are as follows: (1) Size—the size of the particles can facilitate or hinder the application of the nanoparticle as, for instance, larger particle sizes may be more suitable for size-dependent cell uptake or but smaller particles will display reduced steric hindrance when interacting with antibodies [25]; (2) Shape—the shape of the particles will directly influence its uptake into cells, among which rods have highest uptake, followed by spheres, cylinders, and cubes. Additionally, shape will influence the blood circulation as well as the ability to marginate and to bind other elements [26]; (3) Charge—surface charge is an essential feature for blood circulation, particle stability in suspension, and initial absorption to cell membranes [27]; and (4) Ligands—nanoparticle functionalization can mediate protein adsorption, mediate interaction with other molecules, modulate the particle solubility and colloidal stability, and influence the growth rate and shape [24,28].

## 4. Nanotechnology Applications in Sepsis

### 4.1. Diagnosis Devices

The gold standard technique currently employed for diagnosis and pathogen identification is microbiological cultures from biofluids. This technically simple laboratory procedure offers helpful information but is severely hindered by long incubation times, which are unsuitable for emergency diagnostics. Furthermore, a significant proportion of patients with sepsis (approximately 40%) display negative blood cultures, usually due to antibiotic administrations before sampling, low concentrations of pathogen colony-forming units, or atypical pathogens which are not recognized by standard analysis [29,30]. Polymerase-chain-reaction (PCR) detects target pathogen DNA sequences but fails to provide functional information about microbial antibiotic susceptibility. Additionally, this laboratory technique is highly sensitive, and DNA sequences from the host or contaminants that resemble the target sequence could bind the primers used and ultimately produce false positives.

There is a pressing need for technologies that not only allow the rapid and accurate detection of infection but also enable the reliable identification of pathogens and their functional characteristics. This would improve the overall patient outcome by tailoring antimicrobial therapies, reducing the burden of broad-spectrum antimicrobials, and limiting the progression of multidrug-resistant organisms [29]. Nanotechnology-based biosensors display improved sensitivity and processing time while not requiring specialized skills. Nanosized systems also allow the detection of several relevant biomarkers in a rapid and accurate manner, aiding patient diagnosis and ultimately prognosis.

In this section, we describe different nanotechnology-based biosensors, some applications in the detection of clinically relevant biomarkers, as well as innovative approaches currently being developed.

- Electrochemical sensors: These comprise a molecular recognition system and a physicochemical transducer that transforms the chemical responses into an analytical signal [31]. Electrochemical sensors are small devices that exhibit small surface-to-volume ratios and simple immobilization techniques, allowing them to be more rapid, sensitive, selective, and reproducible.
- Immunosensors: These devices use specific antibody–antigen reactions, providing a sensitive and selective tool for the quantification of various biomarkers. Due to the high affinity of the antibodies, signal amplification, high sensitivity, simple fabrication, low cost, reproducibility, and reliability, the application of immunosensors for diagnosis is a growing field of research. These devices usually utilize nanobodies, particles characterized by recombinant variable domains of heavy-chain-only antibodies. These materials exhibit excellent solubility, stability, and specificity, and display quick blood clearance and deep tissue penetration [32].
- Miscellaneous nanosensors: Other diagnostic approaches that have been explored in this field use, for instance, the principles of optical and magnetic resonance properties alongside nanoparticles, allowing the detection of multiple molecules of interest ranging from protein biomarkers to pathogens [14].

#### 4.1.1. Biomarker Detection

A small set of biomarkers have been successfully used in the analytical diagnosis of sepsis, which include CRP, interleukin-6 (IL-6), and procalcitonin (PCT). CRP is a common sepsis biomarker released in response to infection or cytokine stimulation. In healthy individuals, its levels are inferior to 10 mg/L, but it displays an initial rise in 4–6 h after tissue injury and peaks within 24–48 h [33]. It exhibits a good correlation with infection severity and is helpful in the early diagnosis of sepsis patients [34]. IL-6 is a pro-inflammatory cytokine produced in response to infection and tissue injury, significantly contributing to host defense. Lastly, PCT has recently emerged as a sepsis biomarker due to its marked elevation in the presence of bacterial toxins. CRP and IL-6, on the other hand, lack specificity in differentiating bacterial infections from inflammatory responses. The use of these biomarkers is not a new finding but nanotechnology can enhance their quantification in bedside settings, enabling faster and more sensible results without the need for specialized personnel.

Ruppert et al. recently published a report investigating the potential of a quantum dot-labeled lateral flow immunoassay for quantifying CRP and IL-6 [35]. Lateral flow immunoassays are paper-based platforms that detect and quantify analytes in complex mixtures [36]. A liquid sample moves by capillary action through various zones of functionalized polymeric strips, on which molecules that can interact with the analyte are attached [36]. These assays are simple, rapid, robust, and cost-effective, demonstrating the outstanding potential to simplify and accelerate diagnosis. Quantum dots are nanosized particles composed of semiconducting materials such as: cadmium, graphene, silicon, or germanium [37]. When excited by UV-light sources, these particles have characteristic optic and electrical properties that allow them to emit narrow, sharp peaks of a distinct color, serving as a label for bioassays. Using two different quantum dots as labels, one amine- and one carboxyl-modified, allowed the detection of CRP and IL-6 on one test line. The study found that this setup allowed for quantitative readout with an elevated sensitivity. Additionally, with simple adjustments (varying the sample volume, amount of probes applied, use of unlabeled antibodies, and different lateral flow membranes), the method can be made suitable for clinically relevant concentration ranges, establishing this approach as a robust, inexpensive, and rapid point of the care system [35].

Bradley et al. on the other hand have evaluated the effect of nanoparticle size in the detection of IL-6 in a lateral flow device [38]. The study compared the performance of large selenium nanoparticles (between 150 and 310 nm) to that of commercial standard gold nanoparticles (40 nm). For the lateral flow assay, a conjugate pad was coated with anti-IL-6 antibody functionalized nanoparticles (either selenium or gold-based), while the test line was coated with the anti-IL-6 antibody alone. Upon the application of a sample containing IL-6, the selenium or gold nanoparticles bound the analyte. When crossing the test line, the IL-6-conjugated nanoparticles were captured. The color and intensity of the test line were dependent on the size and type of nanoparticle used and proportional to the quantity of particles captured and to the concentration of IL-6. It was found that 150 and 310 nm sized selenium-coated nanoparticles provided superior sensitivity levels at lower concentrations (0–1 ng/mL) compared to 40 nm gold-coated particles. At moderate-to-high concentrations of IL-6 (1–500 ng/mL), the 40 nm gold-coated particles and the 150 nm selenium-coated particles produced more intense test bands. Inherent steric hindrance effects can explain the reduced sensitivity at increasing IL-6 concentrations for larger particles. Larger particles display low surface-to-volume ratios and therefore contain fewer antibody binding sites than the equivalent volume of smaller particles. The 150 nm selenium-coated nanoparticles combined the low limit of detection of the larger particles and the 40 nm gold-coated particle visual intensity in one product. Since the early detection of IL-6 is of greater significance than its detection over a wide concentration range, the 150 nm selenium-coated nanoparticles offer the most desirable detection profile [38].

Last year, Ang et al. published the results of another innovative biosensor, particularly a biofunctionalized magnetic nanoparticle immunoassay of CRP and PCT [39]. The study evaluated the performance of this system in cervicovaginal secretions of pregnant women with preterm pre-labor rupture of membranes to predict early onset neonatal sepsis. In immunomagnetic reduction assays, such as that used in this report, magnetic nanoparticles are homogeneously dispersed in a solution under external alternating current, causing them to oscillate and spin individually. Iron (III) oxide (Fe_3_O_4_) nanoparticles coated in dextran were functionalized with anti-CRP and anti-PCT antibodies (53 and 51 nm, respectively). The functionalization allowed for the clustering of the nanoparticles when bound to the analytes, leading to slower oscillation and spinning. The signal is then obtained by measuring the attenuation in oscillation using a series of equations. This diagnostic strategy allowed for extremely low detection limits, ranging from 10^−4^ ng/mL and 10^−6^ ng/mL in CRP and PCT, respectively [39]. Additionally, the immunomagnetic assays used were of simple pre-processing and allowed for the evaluation of dozens of samples simultaneously.

#### 4.1.2. Other Diagnostic Approaches

Sometimes biomarkers lack specificity, or their levels can be influenced by comorbid conditions. Additionally, they cannot provide information about the pathogen that triggered the inflammatory reaction. Therefore, Abagofi et al. evaluated the efficacy of immunomagnetic separation using vancomycin-conjugated polydopamine-coated magnetic nanoparticles in the detection of Gram-positive bacteria in whole blood [40]. Among the various techniques developed to isolate pathogens from blood samples, such as filtration, centrifugation, sedimentation, and inertial separation, immunomagnetic separation is the most sensitive due to target-specific antibodies [41]. Typically, antibody-conjugated silica-coated magnetic nanoparticles are used to capture a target pathogen. However, an oxide layer is formed when the silica surface is exposed to air or water. Reactive oxygen species formed in this process interact with cell membranes and cause non-specific adsorption and aggregation. Particle aggregation then interferes with PCR and decreases molecular diagnosis sensitivity. To prevent this, the team developed a vancomycin-conjugated polydopamine-coated magnetic nanoparticle. Vancomycin conjugation allows the detection of a broad range of bacterial species, particularly Gram-positive ones. Polydopamine, a highly adaptable polymer, can prevent non-specific binding due to its strong hydrophilicity [40]. Due to its reactivity with amine and thiol groups, it can also be used to immobilize biomolecules on surfaces [42]. The engineered nanoparticles had a diameter of roughly 110 nm. In vitro studies showed that polydopamine-coated nanoparticles did not aggregate in blood samples and exhibited superior Gram-positive bacteria capturing efficiency (~90%), compared to vancomycin-conjugated silica-coated particles (~70%). Additionally, PCR molecular diagnostic with polydopamine-coated nanoparticle preconcentration was superior, displaying a lower limit of detection of 10 CFU/mL, with no significant difference in the preconcentration efficiencies for various bacteria strains [40]. Because the technique does not require any sample pretreatment, bacterial concentration takes roughly 30 min, which is suitable for emergency diagnostic applications. Although the developed particles could only be used to preconcentrate Gram-positive bacteria, the authors speculate that the conjugation of polydopamine-coated magnetic nanoparticles with polymyxin B (PMB) could enable the simultaneous preconcentration of both Gram-positive and negative bacteria.

Similarly, Zhao et al. developed near-infrared fluorescent nanoprobes and magnetic nanoprobes for the rapid capture and detection of bacteria in whole blood [43]. Near-infrared fluorescent probes were prepared by loading indocyanine green into poly(lactic-co-glycolic acid). The particles were then coated with red-blood cell membranes and dibenzocyclooctyne groups with sizes ranging from 120 to 172 nm. Similarly to the fluorescent probes, Fe_3_O_4_ particles were coated with red-blood cell membranes and dibenzocyclooctyne groups and presented a mean size of 343 nm. Coating the probes with red-blood cell membranes reduced the non-specific adsorption with blood cells in the sample. Before capture and detection, bacteria samples were modified with azide groups. The dibenzocyclooctyne groups allowed for rapid conjugation with the azide groups of the bacterial membranes. Due to the probes’ superparamagnetic properties and near-infrared fluorescence, there was rapid and sensitive detection with a fluorescence spectrometer or microscope. The formulation allowed for a detection limit of approximately 4 CFU/mL in less than 2.5 h and was successfully applied to the detection of bacteria in blood samples from patients with sepsis [43].

### 4.2. Treatment Strategies

Unfortunately, current advancements make a single multimodal and specific medicine as an ‘antisepsis’ something beyond the bounds of possibility [44]; therefore, the management of sepsis is multifaceted [45,46]. Current guidelines emphasize the importance of immediate fluid resuscitation and antibiotic administration; however, despite supportive therapy and timely administration, antibiotics are often ineffective and have little impact on lowering patients’ mortality rate [47].

**Figure 2:**
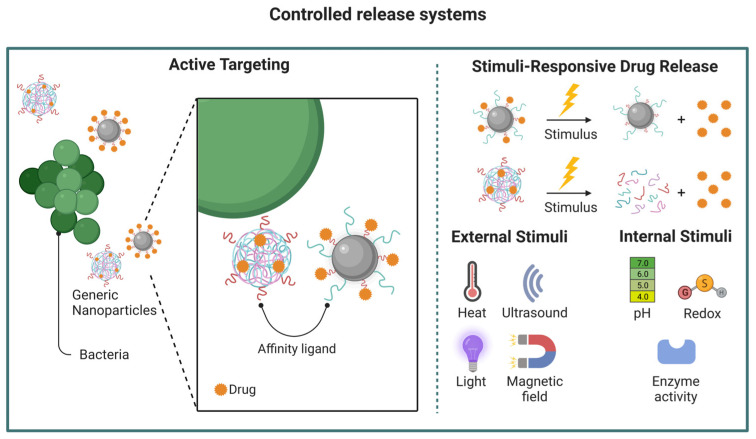
Examples of nanoparticle-mediated drug-delivery. Various delivery platforms can be employed, such as those described in Figure 1. Ideally, these particles can be designed to enable a targeted and controlled release of the active pharmaceutical agent, maximizing the therapeutic effect while minimizing undesired side effects. Image created using BioRender.com.

Due to the ever-evolving increase in drug-resistant pathogens and marked limitations in the development of new antibiotic drugs, the research focus has changed accordingly. Targeted drug delivery, local potency enhancement, and reduced adverse effects have become the main points of focus of antimicrobial research in recent years [14]. Nanotechnology provides benefits beyond tailoring physicochemical features, notably overcoming resistance and preventing its development while minimizing adverse reactions (Figure 2) [14,48]. Nanoparticle formulations can also extend the half-lives of antibiotic drugs by acting as a sustained-release system that enables a reduced frequency of drug administration while improving therapeutic indexes [49,50]. Additionally, many nanomaterials, such as silver and zinc oxide nanoparticles, possess potent inherent antimicrobial activity that can be conveniently used as a treatment adjuvant for antibiotic resistance. This feature is advantageous in inhibiting biofilm generation and targeting intracellular pathogens [51].

#### 4.2.1. Antibiotic-Loaded Nanoformulations

Sepsis guidelines strongly recommend the early and rapid administration of broad-spectrum antibiotics, such as carbapenems [45]. Despite their broad-spectrum antibacterial action with an acceptable safety profile, carbapenems have also been associated with emerging resistance patterns and a short circulation half-life, requiring high-dose administration [14]. A solution was first developed by conjugating carbapenems on the surfaces of gold nanoparticles [52]. Gold nanoparticles are polyhedron structures of gold atoms at the nanoscale, usually spheres that can be conjugated and functionalized with drugs or other molecules. The use of surface-functionalized nanocarriers had been previously successfully explored with metallic atoms such as gold, silver, and iron. Among these, gold nanoparticles are considered particularly advantageous due to their biocompatibility, rapid preparation, and diversity in terms of shapes and sizes, allowing them to be tailored for intra- or extracellular antimicrobial delivery. Gold materials are also efficient loading vehicles and can be configured in various manners: surface covalent bonding, electrostatic adsorption, and drug encapsulation. Shaker et al. evaluated carbapenem-coated gold nanoparticles in an in vitro antibacterial activity assay. An increase in therapeutic efficacy and a decrease in the minimum inhibitory concentration of carbapenem-coated gold nanoparticles were reported compared to carbapenem alone [52]. It also displayed the diffusion-driven release of the drugs from the nanoparticle’s surface, which was prolonged for 48 h. One of the major concerns with these types of particles is their clearance. A recent analysis of gold nanoparticle biodistribution found that they were preferentially accumulated in the liver and spleen [53]. The smaller the diameter of the particle, the broader its distribution was, with minor concentrations present in the kidneys, lungs, hearts, and brains of rodents, specifically mice. The gold content in the liver and spleen did not decrease over time, suggesting poor clearance efficacy through bile ducts. However, a decrease in the gold content in the kidneys could suggest renal clearance, although this phenomenon was only observed for the smallest particles (<8 nm). Due to its accumulation in the liver and kidney, biochemical parameters and histopathology were investigated to determine the toxicity risk. Interestingly, Bailly et al. found that aspartate aminotransferase, alanine aminotransferase, and creatinine levels were comparable to control animal levels, concluding that the accumulation of gold particles did not provoke hepatic or renal toxicity [53]. Additionally, no signs of fibrosis or inflammation were found in the tissues, and normal plasma IL-6 levels suggested the absence of chronic inflammation. Overall, this study demonstrated the safety parameters of these formulations, despite their residual accumulation.

Recently, the novel formulations of antimicrobial-loaded particles emerged, with Mohammed et al. describing an enzyme-responsive biomimetic solid lipid nanoparticle delivery system [54]. This study was directed at hyaluronidase-secreting bacteria. This enzyme can degrade hyaluronic acid, a crucial glycosaminoglycan in many extra- and intracellular functions. Bacterial hyaluronidase has been identified as a significant virulence factor for bacteria species such as *Clostridium perfringens*, *Staphylococcus aureus*, and *Streptococcus pneumoniae*, by enabling them to spread, colonize and form biofilms. Additionally, this enzyme has been recently implicated in the pathogenesis of sepsis by degrading the endothelium glycocalyx, resulting in increased vasculature permeability and promoting systemic inflammation [55]. Besides bacterial hyaluronidase, bacterial lipase is another known bacterial virulence factor, which triggers cell rupture and manipulates the host’s immune system by inhibiting bacterial phagocytosis [56]. In this report, Mohammed et al. evaluated the efficacy of ascorbyl stearate (a vitamin C derivate and potent bacterial hyaluronidase inhibitor) as an adjuvant of a vancomycin tween-80-based lipid nanoparticle delivery system [54]. The addition of ascorbyl stearate was thought to confer both biomimetic and stimuli-responsive properties to the design and enhance its activity against *S. aureus* and methicillin-resistant *S. aureus*. Bacterial lipase was hypothesized to hydrolyze ascorbyl stearate once the nanoparticles reached the infection site, separating the ascorbic acid and stearate moieties. The cleavage would result in a conformational change in the nanoparticle structure, triggering the release of vancomycin in the infection site. Various particles were engineered, with sizes ranging from 93 to 250 nm, depending on ascorbyl stearate to tween-80 ratio. However, for biological activity studies, only 102 nm particles were used. In vitro studies found that this formulation markedly decreased vancomycin’s minimum inhibitory concentration values and allowed for its sustained release. Additionally, it enhanced vancomycin’s bactericide kinetics and allowed for a significant death percentage of treated biofilms. The study by Mohammed et al. showed that the vancomycin–ascorbyl stearate–lipid nanoparticle system has superior antibiotic delivery capabilities, antibacterial activity, and great potential to improve sepsis treatment outcomes [54].

Other reports have evaluated alternative strategies, such as that by Ji et al., which developed a telodendrimer nanocarrier for the delivery of amphotericin B [57]. Amphotericin B is a broad-spectrum antibiotic targeting life-threatening fungal infections. The aggregation of this antibiotic results in significant nephrotoxicity while the monomeric version exhibits much lower cytotoxicity [58]. Some clinically approved liposomal formulations of amphotericin B, such as Fungizone [59,60] (sodium deoxycholate micellar formulation) and AmBiosome [61,62] (composed of α-tocopherol, cholesterol, distearoyl phosphatidylglycerol, and phosphatidylcholine), present reduced toxicity but also reduced drug bioavailability, and its efficacy. This study developed a polyethylene glycol (PEG) dendritic telodendrimer nanocarrier platform to control the aggregation of the antibiotic. Dendritic nanoparticles have hydrophilic exteriors and interiors, responsible for their unimolecular micelle nature [63]. The designed nanoparticles ranged in size but remained small (25–47 nm). In vitro assays found that maintenance of the monomeric form of the antibiotic could be achieved by the introduction of flexible lipid molecules in the particle structure and that these modifications in the formulation abolished the hemolytic effect of the drug even at concentrations of 100 µg/mL. Additionally, antifungal activity was found to be higher, compared to other liposomal formulations, namely Fungizone and AmBiosome. In vivo assays found a sustained drug concentration in the blood and a longer half-life (1.64 h) and did not present severe infusion reactions in mice models after injection. Moreover, in a mouse model of *C. albicans* infection, this telodendrimer nanocarrier showed the most effective antifungal effects, as evidenced by lower CFU counts [57].

Additionally, Alavi et al. evaluated the effects of a PEG coating in a liposome carrier system on the antibacterial effects of nafcillin [64]. This antibiotic is the first-line treatment for methicillin-susceptible *S. aureus* but its use if often limited due to a high cost, which is in need of frequent dose administration and poor tolerability [65,66]. The addition of PEG decreased the nanoparticle size (~240 nm) and resulted in an increased duration of drug release. In vitro assays showed that the loading of nafcillin in nanoparticles resulted in an increase in the antibacterial effects by two- and four-fold for liposome alone, and PEG-coated liposome, respectively. Similarly, PEG-coated liposome was superior to nafcillin or liposome particles against methicillin-susceptible *S. aureus* biofilms in vitro and displayed lower cytotoxicity. In vivo studies evaluated mice weight changes and survival upon challenge with methicillin-susceptible *S. aureus*. PEG-coated liposomes improved animal survival and reduced weight loss, while not eliciting significant liver or kidney toxicity. Overall, the study reported increased efficacy and reduced toxicity in a PEG-coated liposome nanoformulation of nafcillin [64].

#### 4.2.2. Nanoformulation of Antimicrobial Peptides

Antimicrobial peptides (AMPs) have emerged as a novel promising strategy for multidrug-resistant bacterial infections due to their highly rapid bacteriolytic properties [67]. Whereas conventional antibiotics act on intracellular targets, AMP lytic action is mediated by multiple mechanisms, such as interaction with bacterial membranes, leading to physical damage to the bacterial cells [68]. The fast kinetics derived from multiple synergistic pathways significantly reduced the resistance risk and made AMP a unique alternative against multidrug-resistant bacterial infections.

Due to their highly cytotoxic effect, nanomaterials pose as one of the best approaches to the use of these peptides in a directed and controlled fashion, limiting adverse reactions. Yuk et al. evaluated the efficacy of a nanoparticulate system of PMB against Gram-negative bacteria [69]. PMB is a cationic AMP and potent lipopolysaccharide (LPS) adsorbent [70], known to attenuate LPS-induced endotoxemia in mice since 1967 [71]. Despite its potential, PMB exhibits marked nephrotoxicity and neurotoxicity, severely limiting its clinical application [72,73,74]. Yuk et al. aimed to develop a new formulation of this AMP that would enable its safe and systemic use in patients with Gram-negative sepsis. The nanoparticles carrying PMB comprised a tannic acid/Fe^3+^ coordination complex, containing vitamin D as a platform and the conjugation of PMB on the surface along with low-molecular-weight chitosan [69]. The chitosan allowed for attenuating the undesirable contact of PMB with cell membranes without negatively affecting the affinity for LPS. This resulted in an enhanced safety profile that enabled the systemic administration of polymyxin B doses that would have otherwise been lethal. In vivo studies found that the formulation showed maximum efficacy when administered in mice as a mixture with LPS or immediately after LPS [69]. The effectiveness was reduced to 75% and 70% when intravenously administered in mice 2 h after cecal ligation and puncture or LPS challenge, respectively. These survival outcomes are comparable to those previously reported in similar sepsis models. The accumulation of the nanomaterial in the liver showed no sign of hepatotoxicity, consistent with an improved safety profile. Despite the encouraging results, the authors stated that the particle size (~290 nm) hindered its half-time, favoring its accumulation in organs, and that future efforts should be made to optimize this parameter.

Falciani et al. also explored AMP-loaded nanoparticles as inhalation therapy for *Pseudomonas aeruginosa* infections [75]. The system was formulated with dextran nanoparticles, a biologically derived polymer, as carriers for SET-M33. SET-M33 is a synthetic AMP designed in a branched form that confers resistance to degradation and allows for multivalent binding. It has shown efficacy against multiple Gram-negative multidrug-resistant isolates [76] and biofilms [77]. Its effectiveness had been previously established in preclinical infection models, and its safety profile was acceptable. A study describing the SET-M33 mechanism of action demonstrated that, after binding to the bacterial wall LPS, the peptide interacts with the bacterial membrane, embedding itself, destroying the membrane’s function and, eventually, the bacteria itself [78]. This formulation exhibited a very reduced size (18 nm), an acceptable aerosol polydispersity with no tendency to aggregate. and increased lung residence time in rats than the AMP alone, validating its therapeutic inhalation use [75]. It was also effective against *P. aeruginosa* infection in mice, with lower cytotoxicity than SET-M33 alone. Dextran polymers are not usually toxic upon intravenous administration in animals, and the mice used in this study did not display any liver or kidney toxicity after treatment.

Van der Weide et al. studied the AMP AA139, derived from the marine lungworm *Arenicola marina*, against multidrug-resistant *Klebsiella pneumoniae* [79]. AA139 appears to have a dual mode of action involving the direct binding to membrane phospholipids and the interruption of phospholipid transportation pathways, resulting in membrane damage and bacterial cell death [79]. Van der Weide et al. evaluated several nanomedicine formulations, including polymeric nanoparticles and lipid-core micelles. Lipid-core micelles are self-assembling colloidal nanoparticles with a hydrophilic surface and hydrophobic core, where drugs can be entrapped [80]. Both formulations display favorable biocompatibility, nontoxicity, biodistribution, and ease of modification [81,82]. The polymeric nanoparticles used here are dextran-based polymers, with a mean size of 20 nm, attached to AA139 by electrostatic interaction [83]. Lipid-core micelles were engineered with a polyethylene glycosylated distearyl phosphatidyl ethanolamine base and displayed a mean size of 15 nm. The antimicrobial activity of these formulations was assessed by in vitro concentration- and time-dependent bactericidal activity and by in vivo endotracheal aerosolization in rats as a means of direct delivery to the lungs [79]. The in vitro activity of both formulations was comparable to free AA139, suggesting that antimicrobial activity was retained despite the nanoparticle conjugation. Biodistribution studies confirmed a longer lung residence time with polymeric and lipid-core micelles than with AMP alone [79]. Both nanoparticles could be safely administered at a two-fold dose of free AA139. Polymeric nanoparticles displayed a rapid but short-lasting bacterial killing effect, whereas lipid-core micelles showed a slow but sustained effect. These results reflected the difference in biological half-lives between formulations, with polymeric nanoparticles displaying a half-life of roughly 2 h while that of lipid-core micelles was of approximately 3 h. Additionally, lipid-core micelles significantly improved the outcomes when evaluating the efficacy of the formulations by once-daily administration for ten days, with half of the dosage required for polymeric nanoparticles [79].

#### 4.2.3. Other Antimicrobial Nanoformulations

Several alternative strategies for antibacterial therapies have been hypothesized in recent years. According to Zhao et al., most have not considered bacterial extracellular polymeric substances, which remain attached to the bacteria and act as a protective diffusion barrier blocking nanomaterials or drugs [84]. The team speculated that, given that the bacterial extracellular polymeric layer is electrochemically active, the modulation of the electro-microenvironment of biofilms would allow the conduction of antimicrobial treatment. Surface charges can be generated by piezoelectric materials when mechanical stimulation is applied. Thus, the study evaluated the combination of organic piezoelectric nanofiber films with ultrasound stimulation as an antibacterial implant in gastrointestinal (GI) perforation [84]. GI tract perforation is one of the most common causes of sepsis due to the leakage of GI contents into the abdominal cavity. This facilitates bacteria entry into circulation and, subsequently, systemic infection [85]. The piezo implant was composed of poly (vinylidene fluoride-co-trifluoroethylene) films. It was found to successfully enhance the in vitro bactericide efficacy against *E. coli* biofilms and the in vivo inhibition of GI perforation infection in rats [84].

The immune paralysis associated with sepsis predisposes critically ill patients to secondary infection [14]. Beyond antibiotic therapy and supportive measurements, these patients require specific strategies directed to restore the function of the immune system. Some recent nanoformulations have focused on targeting inflammation rather than aiming to kill bacteria. One such application was recently developed by Chen et al. [86]. The cytokine storm that follows the initial triggering of PAMPs or DAMPs leads to generalized pyroptosis [87,88]. Usually, pyroptosis plays a positive role in the immunomodulatory process, but when the host is undergoing severe infection, it can be hyperactivated and exacerbate inflammation. Current sepsis treatment strategies are mainly anti-infection; however, an effective way to prevent sepsis installment or manage multi-organ damage resulting from excessive inflammatory damage is lacking to date. To design a nanoparticle that would inhibit pyroptosis, Chen et al. developed tetrahedral framework nucleic acids [86]. These novel nanomaterials have been shown to have enhanced cell endocytosis properties and tissue permeability, rendering them suitable for biomedical applications. Previous studies have also demonstrated that these structures exhibit anti-inflammatory and antioxidant capabilities while maintaining a good biosafety profile [89]. The tetrahedral framework nucleic acid were assembled from four single-stranded sequence-specific DNA fragments with a mean size of 17 nm. The in vitro and in vivo assays performed by Chen et al. confirmed the protective effect of the tetrahedral framework nucleic acids against macrophages under LPS stimulation and their preventive effect on reducing the inflammatory response in septic mice [86]. These results indicate the potential of pyroptosis inhibition in managing sepsis and the usefulness of these formulations.

## 5. Conclusions and Future Directions

Nanotechnology is a complex field that has shown great promise in biomedical applications. Nanomaterials can be engineered to yield specific chemical, physical, and surface properties that more adequately achieve the desired biological effect. They can significantly vary in size, shape, composition, and overall effect. The use of organic vs. inorganic materials greatly impacts biocompatibility, toxicity, immunogenicity, stability, the modulation and functionalization process, batch-to-batch variability, and tracking capabilities. Recent progress in terms of nanotechnological applications in the field of sepsis have allowed for the development of new diagnosis and treatment strategies. Nanotechnology-based biosensors are small devices that exhibit small surface-to-volume ratios and simple immobilization techniques, allowing them to be more rapid, sensitive, selective, and reproducible. The use of nanoparticles was found to facilitate the quantification of various sepsis biomarkers (CRP, IL-6, and PCT) as well as the detection of both Gram-positive and Gram-negative bacteria in whole blood samples. Additionally, nanoparticles have allowed for targeted drug delivery, the inhibition of biofilms, improvements in local antimicrobial activity, and reduced cytotoxicity. They have improved the half-life of several drugs, making them more attractive options for clinical use. Nanotechnology has also allowed to take advantage of the extreme antimicrobial potential of AMP while minimizing their adverse effects. Some nanomaterials even possess potent inherent antimicrobial activity that can be used as a treatment adjuvant for antibiotic resistance.

Despite the positive results and exciting novel approaches, clinical trials fully embracing these developments are still limited. After nearly thirty years of fundamental studies, a solid foundation has been achieved, and a different focus should be selected. It is imperative to prioritize the application of these strategies from bench to bedside, where patients can fully benefit from scientific advancement. Incorporating physicians in teams can help target diagnostic and therapeutic approaches according to the current challenges in clinical practice. Close articulation will allow for the development of biobanks that will enable the more precise determination of device reliability and refine the technology for patient use. In that sense, it is also essential to share information and results in a manner that is accessible to everyone that may be interested in the subject. Nanotechnological applications, whether regarding devices or therapeutical approaches, are often presented in very technical formats, ultimately hampering their translation. Accordingly, review papers that aim to simplify the technical components of the technology and highlight the intended use cases and their advantages are key to fostering communication and attract more diverse areas of studies to research teams. Altogether, we envision synergistic collaborations as a means of streamlining innovative solutions, particularly in the field of nanomedicine.

## Data Availability

Not applicable.
